# MET exon 14 skipping mutation is a hepatocyte growth factor (HGF)‐dependent oncogenic driver *in vitro* and in humanised *HGF* knock‐in mice

**DOI:** 10.1002/1878-0261.13397

**Published:** 2023-07-14

**Authors:** Marie Fernandes, Brynna Hoggard, Philippe Jamme, Sonia Paget, Marie‐José Truong, Valérie Grégoire, Audrey Vinchent, Clotilde Descarpentries, Angela Morabito, Justas Stanislovas, Enoir Farage, Jean‐Pascal Meneboo, Shéhérazade Sebda, Katia Bouchekioua‐Bouzaghou, Marie Nollet, Sarah Humez, Timothy Perera, Paul Fromme, Luca Grumolato, Martin Figeac, Marie‐Christine Copin, David Tulasne, Alexis B. Cortot, Stéphanie Kermorgant, Zoulika Kherrouche

**Affiliations:** ^1^ Univ. Lille, CNRS, Inserm, CHU Lille Institut Pasteur de Lille, UMR9020 – UMR1277 - Canther – Cancer Heterogeneity, Plasticity and Resistance to Therapies France; ^2^ Barts Cancer Institute Queen Mary University of London UK; ^3^ Univ Lille Department of Pathology, CHU Lille France; ^4^ Univ. Lille, Molecular Biology, Hormonology Metabolism Nutrition Oncology, CHU Lille France; ^5^ Univ. Lille, CNRS, Inserm, CHU Lille, Institut Pasteur de Lille, US 41 - UAR 2014 - PLBS, Lille France; ^6^ OCTIMET Oncology NV Beerse Belgium; ^7^ Department of Mechanical Engineering University College London UK; ^8^ Univ Rouen Normandie, Inserm, NorDiC UMR 1239, 76000 Rouen France; ^9^ Univ. Lille Thoracic Oncology Department, CHU Lille France

**Keywords:** hepatocyte growth factor, lung cancer, preclinical models, targeted therapies, transcriptomic, tyrosine kinase receptor

## Abstract

Exon skipping mutations of the MET receptor tyrosine kinase (METex14), increasingly reported in cancers, occur in 3–4% of non–small‐cell lung cancer (NSCLC). Only 50% of patients have a beneficial response to treatment with MET‐tyrosine kinase inhibitors (TKIs), underlying the need to understand the mechanism of METex14 oncogenicity and sensitivity to TKIs. Whether METex14 is a driver mutation and whether it requires hepatocyte growth factor (HGF) for its oncogenicity in a range of *in vitro* functions and *in vivo* has not been fully elucidated from previous preclinical models. Using CRISPR/Cas9, we developed a METex14/WT isogenic model in nontransformed human lung cells and report that the METex14 single alteration was sufficient to drive MET‐dependent *in vitro* anchorage‐independent survival and motility and *in vivo* tumorigenesis, sensitising tumours to MET‐TKIs. However, we also show that human HGF (hHGF) is required, as demonstrated *in vivo* using a humanised HGF knock‐in strain of mice and further detected in tumour cells of METex14 NSCLC patient samples. Our results also suggest that METex14 oncogenicity is not a consequence of an escape from degradation in our cell model. Thus, we developed a valuable model for preclinical studies and present results that have potential clinical implication.

## Graphical abstract

Using CRISPR/Cas9, we developed a METex14/WT isogenic model in nontransformed human lung cells, thus without the confounding effects of other oncogenic alteration. METex14 single alteration was sufficient but required HGF to drive MET‐dependent sustained phosphorylation and signalling, enhanced cell motility, anchorage‐independent survival, changes in gene expression and *in vivo* tumorigenesis in humanised *HGF* knock‐in mice, sensitising tumours to MET‐TKIs.

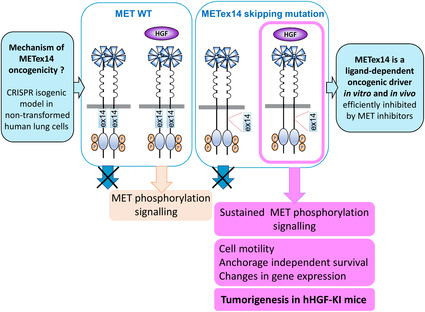

**Abbreviations**
ADC: adenocarcinoma
CGH: comparative genomic hybridization
DMEM: Dulbecco's modified Eagle medium
DMSO: Dimethyl sulfoxide
DNA: deoxyribonucleic acid
EGFR: Epidermal Growth Factor Receptor
FDA: U.S. Food and Drug Administration
FISH: fluorescence *in situ* hybridization
FFPE: formalin fixed paraffin embedded
GEO: Gene Expression Omnibus
GO: Gene Ontology
HGF: hepatocyte growth factor
IHC: immunohistochemistry
METex14: MET exon 14 splicing variant
NGS: Next‐Generation Sequencing
NSCLC: non–small‐cell lung cancer
RPMI: Roswell Park Memorial Institute‐1640
RNA: ribonucleic acid
RTK: receptor tyrosine kinase
RT‐PCR: reverse transcription polymerase chain reaction
SD: standard deviation
STR: short tandem repeat

## 1 Introduction

Targeted therapies against receptor tyrosine kinases (RTKs) are currently used with success in cancers displaying clear oncogene addiction, such as in epidermal growth factor receptor (EGFR)‐mutated lung cancers [1]. Most commonly targeted EGFR mutations occur in the kinase domain and lead to constitutive activation, resulting in oncogene‐dependent cell growth and survival [2].

MET, an RTK found predominantly in cells of epithelial origin, is activated by its stromal ligand, hepatocyte growth factor (HGF). Deregulation leading to constitutive activation has been described in several cancers. These can be caused by activating mutations in the kinase domain or, more commonly, *MET* gene amplification [3, 4, 5], and can lead to HGF‐independent cellular proliferation and tumour growth [6, 7, 8, 9]. There is also evidence that *MET* amplification in some gastric and lung cancers can predict response to targeted therapy [10, 11].

Recently, a set of alterations have been identified in several cancers including non–small‐cell lung cancer (NSCLC) affecting the splice donor or acceptor sites of MET exon 14 [3]. These alterations include point mutations, deletions, insertions and complex mutations which all result in the in‐frame skipping of exon 14, and deletion of the 47 amino acid juxtamembrane domain (METex14). These mutations have been reported in lung adenocarcinomas (2.6–3.2%) and pulmonary sarcomatoid tumours (2.6–31.8%) [12, 13, 14, 15, 16, 17]. METex14 has also been observed in other tumour types, including gastric cancers (7.1%), colorectal cancers (0–9.3%), brain gliomas (0.4–14%) and other malignancies [13, 18, 19].

Exon 14 contains important residues involved in receptor downregulation [20, 21, 22, 23]. The best characterised is the binding site for the E3 ubiquitin ligase c‐Cbl, tyrosine 1003 (Y1003), which is important for receptor ubiquitination and degradation. Early studies have indicated that juxtamembrane domain loss could lead to MET deregulation and tumorigenesis [24, 25].

MET tyrosine kinase inhibitors (MET‐TKIs) are already being evaluated in patients harbouring exon 14 skipping [26, 27]. The VISION clinical trial evaluating tepotinib in METex14 NSCLC patients has reported an objective response rate (ORR) of about 50% [28], while the GEOMETRY mono‐1 trial evaluating capmatinib has reported an ORR of 41% and 68% in pretreated and treatment‐naïve METex14 patients, respectively [29]. Both of these MET‐TKIs have been approved by the FDA for METex14 NSCLC patients. However, the ORR and progression‐free survival achieved with these MET‐TKIs appear to be lower than those achieved with TKIs in other oncogene‐addicted NSCLC. Thus, it is increasingly important to better understand the mechanism of METex14 oncogenicity and sensitivity to TKIs.

Whether METex14 is a driver mutation has remained elusive. Many studies have used preclinical models with transformed cell lines [9, 25, 26, 30, 31, 32]. In such situations, it is unclear whether MET oncogenicity is driven by METex14 or the concomitant alteration or both. In addition, several cell models overexpress MET in conjunction with exon 14 skipping [13, 24, 25]. MET amplification or overexpression on their own can lead to MET constitutive activation, due to ligand‐independent receptor dimerization [33, 34]. Therefore, it can not be determined in these models whether MET oncogenicity is driven by METex14 or its overexpression. Consistent with this, whether HGF is required for METex14 activation and oncogenicity or whether METex14 is constitutively phosphorylated has remained unclear. Furthermore, many *in vivo* studies have utilised xenografts of human cell lines in various mice models such as SCID or nude. As murine HGF can not activate human MET in the grafted cells [35, 36], and as most cell models used were transformed, often with overexpression of METex14, the mechanisms of such tumorigenesis models and thus of METex14 oncogenicity *in vivo* and the specificity of the response to MET‐TKIs are not fully elucidated [31, 37]. Therefore, appropriate preclinical models, allowing dissection of the influence of METex14 on its own, with no concomitant genetic alteration, in both the absence and the presence of the ligand, are required.

Using CRISPR/Cas9, we developed an isogenic human lung cell model expressing METex14. With this model, we investigated the ability of exon 14 skipping to transform cells *in vitro* without the confounding effects of *MET* amplification or overexpression and to drive tumorigenesis *in vivo* with or without the presence of human HGF, using NSG‐hHGF knock‐in mice. We demonstrate that the METex14 mutation is a driving mutation, but which requires HGF for its oncogenicity *in vitro* and *in vivo*. Interestingly, we have also observed that METex14 oncogenicity is not coupled to an escape from degradation in our nontransformed cell model. Our results suggest that tumours bearing METex14 mutation, on its own with no other genetic alteration, will likely display response to MET‐targeted therapies if HGF is both present and accessible to the cancer cells.

## 2 Materials and methods

### 2.1 Patients

Clinical and molecular data were collected retrospectively at CHU Lille. In accordance with the European general data protection regulation for a retrospective noninterventional research, a data processing declaration was made to the data protection officer of the Lille University Hospital (no. 273), a privacy impact assessment was carried out and informed information was given to living patients in the context of this project; for patients who were not able to give consent for this project, nonopposition was sought in the medical records.

### 2.2 Cell lines

All cell lines were cultured in media with 10% foetal bovine serum from Eurobio‐scientific and maintained in humidified incubators at 37 °C and 5% CO_2_. Mycoplasma tests were routinely performed using the MycoAlert mycoplasma detection kit (Lonza, Basel, Switzerland). The nontumorigenic 16HBE14o‐ (RRID:CVCL_0112) (16HBE) cell line developed by immortalising primary human bronchial epithelial cell with SV40 Large T antigen, was a kind gift from Pr Dieter Gruenert and cultured in MEM [38]. The non–small‐cell lung cancer cell line cell lines H226 and H596 were obtained from Dr Kong‐Beltran [25]. H226 cells (RRID:CVCL_1544) expressing endogenous MET WT and H596 cells (RRID:CVCL_1571), an established lung adenocarcinoma cell line with a homozygous point mutation leading to exon 14 skipping and concurrent activating PIK3CA mutation, were both cultured in RPMI1640. The MRC5 fibroblasts (RRID:CVCL_0440) were cultured in MEM.

MET exon 14 skipping was introduced in 16HBE cells by electroporation with px459V2 vector (Addgene #62988, Watertown, MA, USA) containing cDNA coding for Cas9 enzyme and sgRNA sequence (5′‐TACCGAGCTACTTTTCCAGA‐3′) targeting the exon 14 splice donor region. After 24 h, cells were selected in 1 μg·mL^−1^ puromycin and isolated by limit dilution in 96‐well plates. Screening was performed by western blot and PCR using methods described previously [39]. The parental cell line and the METex14 expressing subclones have been authenticated using short tandem repeat profiling in 2021 or 2022 (Eurofins Genomics, Ebersberg, Germany). In addition, METex14 mutation and the absence of alteration in other oncogenic drivers were validated by NGS sequencing using the CLAPv1 targeted NGS panel using methods described previously [39].

### 2.3 Transcriptomic analysis

Cells (750 000) were plated in 10‐cm dishes in full media. After 24 h, cells were serum‐starved 2 h and treated for 24 h with HGF. Total RNA was extracted (Nucleospin RNA, Macherey‐Nagel, Düren, Germany). Genomic DNA traces were removed by DNAseI treatment. Experiment was performed in four replicates each separated by one‐week culture using methods described previously [40, 41]. Briefly, total RNA yield and quality were evaluated on the Nanodrop 2000C system (ThermoFisher Scientific, Waltham, MA, USA) and further assessed on the Agilent 2100 bioanalyzer (Agilent Technologies, Santa Clara, CA, USA). One colour whole Human (072363_D_F_20150612) 60‐mer oligonucleotides 8 × 60 k microarrays (Agilent Technologies) were used to analyse gene expression. cRNA labelling, hybridisation and detection were carried out according to the supplier's instructions (Agilent Technologies). For each microarray, Cyanine 3‐labelled cRNA were synthesised with the low input QuickAmp labelling kit from 50 ng of total RNA. RNA Spike‐In was added to all tubes and used as positive controls of labelling and amplification steps. The labelled cRNA were purified, and 600 ng of each cRNA was then hybridised and washed following the manufacturer's instructions. Microarrays were scanned on an Agilent G2505C scanner and data extracted using the agilent feature extraction Software (FE version 10.7.3.1). Statistical comparisons, filtering and figures were achieved with limma r package (R3.5.1, limma 3.38.3).

### 2.4 Reagents and chemicals

MET inhibitors crizotinib and capmatinib from Selleck Chemicals (Houston, TX, USA) were prepared in DMSO. The MET‐TKI OMO‐1 from OCTIMET was suspended in DMSO for cell treatment, and a vehicle containing 0.5% methylcellulose and 0.1% Tween80 in water, sonicated and brought to a pH of 2.5–3.5 for *in vivo* use.

### 2.5 Western blot and cell signalling experiments

Cells were grown in plates for 24 (H226 and H596) or 48 h (16HBE‐WT and 16HBE‐ex14) and serum‐starved either overnight or 1 h before treatment, as indicated in legends, with recombinant HGF (Selleck Chemicals or Miltenyi, Bergish Gladbach, Germany or R&D System, Minneapolis, MN, USA) for the times indicated. Proteins were resolved on polyacrylamide gels and transferred to nitrocellulose (GE Healthcare, Chicago, IL, USA) or Immobilon‐P (Merck Millipore, Burlington, MA, USA) membranes. The membranes were blocked in Casein buffer (0.2% casein in PBS with 0.1% Tween20) 1 h at room temperature and probed in BSA buffer (5% Bovine serum albumine in PBS with 0.1% sodium azide) overnight at 4 °C, with the following antibodies, used at 1 : 1000 dilution: MET (CVD13, Invitrogen 71‐8000, Carlsbad, CA, USA), MET (Cell Signalling #3148, Danvers, MA, USA), phospho‐MET (Tyr1234/1235, Cell Signalling #3126), phospho‐AKT (S473, Cell Signalling #4060), AKT (Cell Signalling #9272; Santa Cruz sc‐8312, Dallas, TX, USA), phospho‐ERK1/2 (Cell Signalling #9106, #4370), ERK1/2 (Cell Signalling #9102), ERK2 (Santa Cruz sc‐154) and HSC70 (Santa Cruz sc‐7298). Peroxidase‐coupled secondary antibodies from Jackson ImmunoResearch Laboratories, West Grove, PA, USA (anti‐mouse 115‐0350146; anti‐rabbit 711‐0350152) or BioRad (anti‐mouse 170‐6516; anti‐rabbit 170‐6515) were used at 1 : 10 000 and 1 : 2000 dilution, respectively. Proteins were detected using Amersham‐enhanced chemiluminescence (ECL) detection agents (GE Healthcare) and developed on X‐ray film or using the Amersham Imager 600 (GE Healthcare) or the Gel Doc Systems (Thermofisher). Densitometry was quantified using imagej (National Institutes of Health, Bethesda, MD, USA).

### 2.6 Cell number and cell viability assay in anchorage independent conditions

Cells were seeded (5000 cells) in 96‐well plates (Corning, NewYork, NY, USA) in complete media containing Nuclight Red fluorescent dye for live‐cell nuclei staining (Essen Bioscience, Ann Arbor, MI, USA) and supplemented or not with 20 ng·mL^−1^ HGF. The number of fluorescent nuclei was quantified over time using an Incucyte apparatus.

For anchorage‐independent spheroid viability assay, cells were seeded (2000 cells) in 96‐well ultra‐low attachment plates (Corning) in media containing 0.1% FBS and treatments. Fresh media and treatments were added every 48–72 h (20 μL per well). After 14 days, cell viability was quantified with Alamar Blue reagent (Invitrogen) and fluorescence measured on a Fluostar Optima plate reader (BMG Labtech, Ortenberg, Germany) with 560/590 (ex/em) wavelength filter settings.

### 2.7 Cell colony formation assay and measure of distance between cells

Colony formation were performed on 13 mm coverslips. Cells were seeded at low densities (500 cells per coverslip) and treated every 2–3 days, without or with HGF in presence of DMSO or MET‐TKIs, as indicated. After 6 days, cells were fixed using 4% paraformaldehyde. Plasma membranes and nuclei were labelled with WGA‐488 (10 μg·mL^−1^; Thermofisher) and DAPI. Images of random colonies were acquired with a Zeiss LSM 510 or 710 at 40×. cellprofiler was used to automatically detect nuclei and average distances from the centroid of each nucleus to the second nearest nucleus were calculated for each condition.

### 2.8 Transwell migration assay

Cells were labelled for 1 h with 10 μg·mL^−1^ DilC12(3) fluorescent dye (Corning) and 40 000 cells seeded in complete medium onto a 24‐well FluoroBlok PET permeable support system (Corning). After 24‐h incubation, serum‐free medium was added to the upper chambers, and the lower chambers were filled with 20 ng·mL^−1^ HGF in serum‐free medium as attracting factor. The fluorescence of migrating cells was measured over time with a Fluostar Optima plate reader with 549/565 (ex/em) wavelength filter settings.

### 2.9 Spheroid invasion assay

A protocol modified from [42] was used. Spheres were formed in 2.5% (v/v) methylcellulose 4000 cP (Sigma‐Aldrich, Saint‐Louis, MO, USA) hanging droplets using 1000 cells total in a 2 : 1 ratio MRC5 fibroblasts: 16HBE‐WT or ‐ex14 cells. Spheres were collected 24 h later and suspended in organotypic mixture (10.5 volumes high concentration Collagen (354249, Corning, 2 mg·mL^−1^ final concentration), 7 volumes Matrigel, 1 volume HEPES (1 m, pH 7.5, H7006, Sigma‐Aldrich) and 21.5 volumes relevant cell culture medium, with 1 m NaOH added dropwise to neutralise the pH), before being seeded into wells of a 96‐well plate. Culture medium containing relevant treatments was added on top of gels once set. Gels were imaged using an Axiovert 135 (Carl Zeiss MicroImaging LLC, Ortenberg, Germany) camera and percentage invasive area quantified using imagej (National Institutes of Health), using the following equation:

$$\%\text{invasive area}=\left(\left(\text{total area}-\text{central area}\right)/\text{central area}\right)\times 100.$$

### 2.10 Immunohistochemistry

MET and HGF stainings were processed on the Benchmark ULTRA automated system (Ventana Roche, Tucson, Arizona) according to the manufacturer's protocols. Briefly, after the tissue sections were deparaffinised with EZ prep (Ventana), heat‐induced epitope retrieval with CC1 (Ventana) was performed with an incubation time of 64 min and the slides were incubated with primary antibodies against MET (clone SP44, Ventana, ready‐to‐use dilution incubated 16 min) and HGF (clone 4C12.1, LS Bio, at 1/100 dilution incubated 32 min). For MET, a score IHC 3+ tumour was used as a positive control staining [43] and a tonsil tissue sample for HGF. Immunoreactions were detected by the Ultraview DAB Universal detection kit for MET and by the Optiview DAB Universal detection kit for HGF, followed by counterstaining with Hematoxylin II and Bluing reagent (Ventana). For HGF, a semiquantitative scoring was performed by multiplying the percentage of positive stained cells by the intensity of labelling evaluated by pathologist visual scoring of staining on a scale of 0–3+.

### 2.11 Mice experimentations and approval

The project and experimental protocols received an ethical approval by the French Committee on Animal Experimentation and the Ministry of Education and Research (approval number 19253‐201903191709966 v1). All experiments were performed in accordance with relevant guidelines and regulations. Mice were bred under SOPF conditions at the Animal Research Laboratory of Institut Pasteur de Lille. Experiments were performed in an isolator with six mice housed in M‐BTM cage (Innovive) and allowed to eat and drink *ad libitum*. Xenografts were performed in the NOD.*scid*.Il2Rγc (NSG) mice with a humanised HGF knock‐in allele (KI‐huHGF) or in the control NSG mice (Jackson Laboratory), in both female and male group of mice without noticing any difference. 16HBE cells (2 × 10^6^) in PBS were injected subcutaneously into both flanks of 6‐ to 9‐week‐old mice. Tumours were palpated and measured with callipers at least two times a week. When tumours reached 100 mm^3^ in volume, mice received 48 mg·kg^−1^ OMO‐1 or the vehicle as a control by oral gavage once daily. In a second experiment, mice were treated 30 days after injection with 50 mg·kg^−1^ crizotinib or the vehicle The tumour volume (*V*) was calculated with the formula *V* = 0.5 × (*L* × *W* ^2^). Animals were culled before tumours reached 1000 mm^3^.

### 2.12 Statistics

All results are expressed as mean ± SD. According to their distribution, quantitative variables were compared with a *t*‐test, Mann–Whitney test or two‐way ANOVA. Two‐way ANOVA was performed to compare the entire curves of the tumour growth analyses in subcutaneous xenograft models. All statistical testing was conducted at the two‐tailed *α* level of 0.05. Data were analysed with the graphpad prism (San Diego, CA, USA) software version 9.

## 3 Results

### 3.1 Activation of METex14 is dependent on HGF and results in sustained downstream signalling

We developed an isogenic cell model to investigate METex14 without the confounding effects of MET amplification or overexpression. Using CRISPR/Cas9, we introduced MET exon 14 skipping in 16HBE cells (16HBE‐ex14), an immortalised, nontumorigenic human bronchial epithelial cell line. METex14 expression was validated in two clones, 16HBE‐ex14 clone F and clone 7, by RT‐PCR (Fig. S1) and the absence of off‐target alterations was investigated by next generation sequencing with the panel CLAPv1 [39]. In parallel, we analysed the lung adenocarcinoma cell lines H226, expressing endogenous WT MET, and H596 with endogenous exon 14 skipping.

**Fig. 1:**
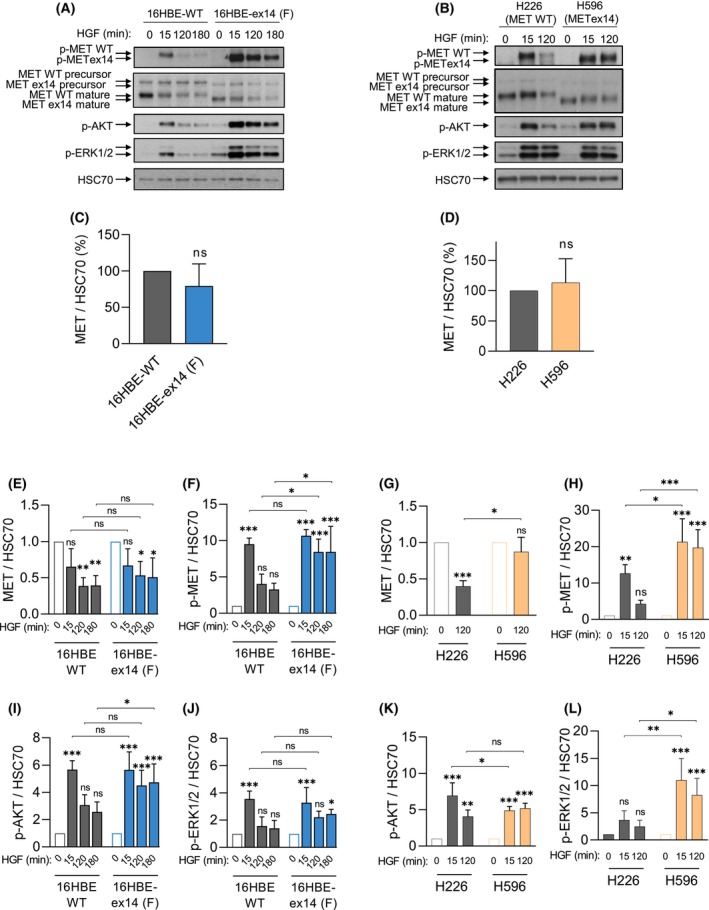
Activation of exon 14 spliced MET is dependent on HGF, and results in sustained downstream signalling. (A) 16HBE cells expressing either WT MET or METex14, following CRISPR/Cas9 editing and (B) H226, expressing MET WT and H596 cells expressing endogenous MET ex14, were grown for 48 h (A) and 24 h (B) and serum‐starved 1 h before treatment with 50 ng·mL^−1^ HGF for the times indicated. For each condition, whole cell lysates were resolved by SDS/PAGE and analysed by western blotting with the indicated antibodies. Arrows indicate MET WT or ex14 precursor or beta‐chain mature forms. Quantification by densitometry, normalised to a loading control (HSC70) is represented as fold change of MET expression in unstimulated cells (C, D), and of MET expression (E, G) or MET, AKT and ERK1/2 phosphorylation (F, H–L) levels upon HGF activation in each cell line. Data are means of three experiments, −/+ SD, Two‐way ANOVA test, *P < 0.05, **P < 0.01, ***P < 0.001, ns, not significant.

METex14 protein levels were not significantly different to MET WT in all cell model systems (Fig. 1A–D and Fig. S2A). When cell lines were incubated with HGF for 120 or 180 min, protein levels of the mature beta chain of MET WT exhibited a significant reduction compared with unstimulated cells in both models (Fig. 1A,B,E,G and Fig. S2A). Upon HGF activation, MET is quickly internalised into the cell and progressively degraded [44, 45]. Therefore, reduced cellular levels of MET are often observed after activation with HGF.

In H596 cells, METex14 protein levels were not significantly different after 120 min of HGF stimulation, compared with no stimulation (Fig. 1B,G). This observed stability of METex14 is consistent with previous reports that the loss of exon 14 prevents binding to CBL and subsequent protein ubiquitination and degradation of MET [25, 46]. However, in 16HBE cells, METex14 was significantly degraded after 120 and 180 min of HGF activation. Although it displayed a trend of increased stability versus MET WT in the same cells, the differences were not statistically different (Fig. 1A,E).

We also measured the signalling response to the ligand stimulation in all our cell lines. Similarly to the WT receptor, METex14 responded to increasing concentrations of HGF (Fig. S3). Both WT cells and METex14 cells were found to be dependent on HGF for MET activation in both paired cell models, and exhibited significantly high levels of phosphorylated MET 15 min after HGF stimulation compared with no stimulation. In both cell systems, while MET WT phosphorylation significantly decreased with longer incubation times, METex14 phosphorylation was significantly sustained up to 3 h (Fig. 1A,B,F,H and Figs S2A and S4A–F). A sustained activation of AKT or ERK1/2 was observed in response to HGF stimulation in METex14 cells (Fig. 1I–L and Figs S2A, S4A and S4G,H).

These results, obtained in the cells expressing endogenous MET WT and introduced (CRISPR/Cas9) MET Ex14 deletion clones F and 7, indicate that, in the presence of HGF, METex14 phosphorylation and downstream signalling are sustained compared with MET WT, and this independently from METex14 stability.

### 3.2 Upon HGF stimulation, METex14 promotes cell motility and anchorage independent survival

To assess whether METex14 is a driver mutation for a range of cell functions, we measured over time cell numbers and cell motility and performed 3D survival and 3D invasion assays, with or without HGF, in our 16HBE isogenic CRISPR cells expressing MET WT or ex14 (clone F and/or 7).

**Fig. 2:**
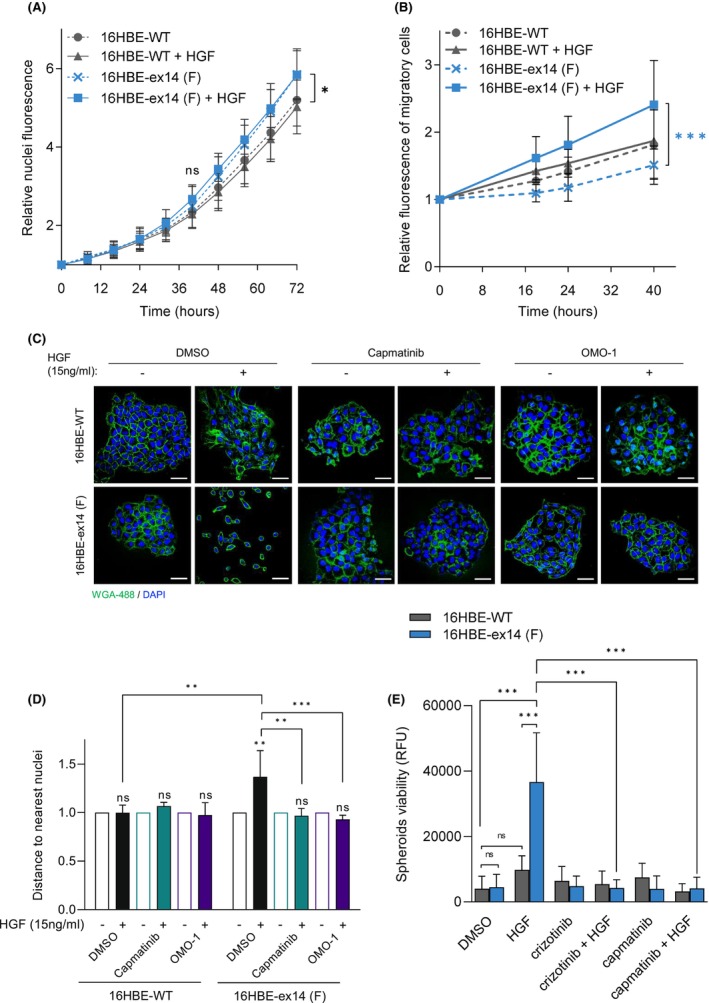
Transforming capacities of 16HBE‐ex14 cells are dependent on HGF stimulation *in vitro*. (A) Real‐time cell numbers of adherent 16HBE cell lines, stimulated or not by 20 ng·mL^−1^ of HGF, was determined through viable nuclei labelling and live imaging in an IncuCyte ZOOM over 72 h. (B) Migration of DilC12‐labelled 16HBE cells was determined in a transwell assay, with or without HGF (20 ng·mL^−1^) in the medium of the lower chamber. Fluorescence of migrating cells was measured over time and expressed as relative mean migration. (C, D) 16HBE cells were seeded at a low density on coverslips in full media and stimulated or not by 15 ng·mL^−1^ of HGF in the presence of DMSO, capmatinib or OMO‐1 (1 μm). (C) Representative confocal sections of cells fixed at day 6 are shown (scale bar: 50 μm). (D) Distances from the centroid of each nucleus to the second nearest nucleus represented as fold change with HGF over without HGF. (E) The viability of non‐adherent spheroids of 16HBE cells seeded in ultra‐low attachment plates with low‐serum media containing HGF (20 ng·mL^−1^), crizotinib (1 μm) or capmatinib (1 μm), was evaluated after 14 days by reduction of resazurin (Alamar Blue reagent). All data are means of three experiments with at least three wells per condition, Two‐way ANOVA test −/+ SD, *P < 0.05, **P < 0.01, ***P < 0.001, ns, not significant.

A 72 h live‐cell assay performed in 2D adherent cultures indicated that while 16HBE‐ex14 cell number was slightly higher than 16HBE‐WT over time, stimulation with HGF had no effect in either cells (Fig. 2A).

Cell motility was assessed using Transwell filters. Only 16HBE‐ex14 cells demonstrated significantly improved migratory ability upon HGF stimulation in the conditions tested (Fig. 2B and Fig. S2B).

To promote colony formation, cells were grown at low density in adherent conditions, both with and without HGF. After 6 days in the presence of HGF, 16HBE‐ex14 cells formed less‐compact colonies than WT cells with noticeable gaps between the cells (Fig. 2C), which were measured (see Section 2) and were significantly bigger (Fig. 2D). However, following the treatment with the MET‐TKIs capmatinib or OMO‐1, the distance between the cells within colonies was reduced to WT levels.

Cell viability in 3D anchorage‐independent and low‐serum conditions was measured. Activation with HGF significantly increased the viability of 16HBE‐ex14 cells compared with WT cells, which was efficiently blocked with the addition of the MET‐TKIs crizotinib or capmatinib (Fig. 2E).

We also developed a spheroid model, using HGF‐secreting fibroblasts [45] to study the model's invasion capacity in 3D. 16HBE cells were co‐cultured with MRC5 fibroblasts within methylcellulose hanging drops to form spheroids, before being placed into collagen:Matrigel hydrogels. In control cultures with no MRC5, no spheroids were formed (data not shown). However, spheres of MRC5 and epithelial cells exhibited collective invasion which was significantly increased with 16HBE‐ex14 cells, compared with WT. The invasion was reduced upon capmatinib treatment (Fig. S2D).

These results, obtained in isogeneic cells expressing MET WT or ex14 (clone F or 7) indicate that the METex14 alteration on its own, without the requirement of other genetic alterations or MET overexpression, is sufficient to transform cells, especially when cultured in a 3D setting. However, HGF is required, suggesting METex14 is an HGF‐dependent driver mutation.

### 3.3 Transcriptomic analysis of HGF‐activated METex14 revealed regulation of genes involved in extracellular matrix and structure organisation

**Fig. 3:**
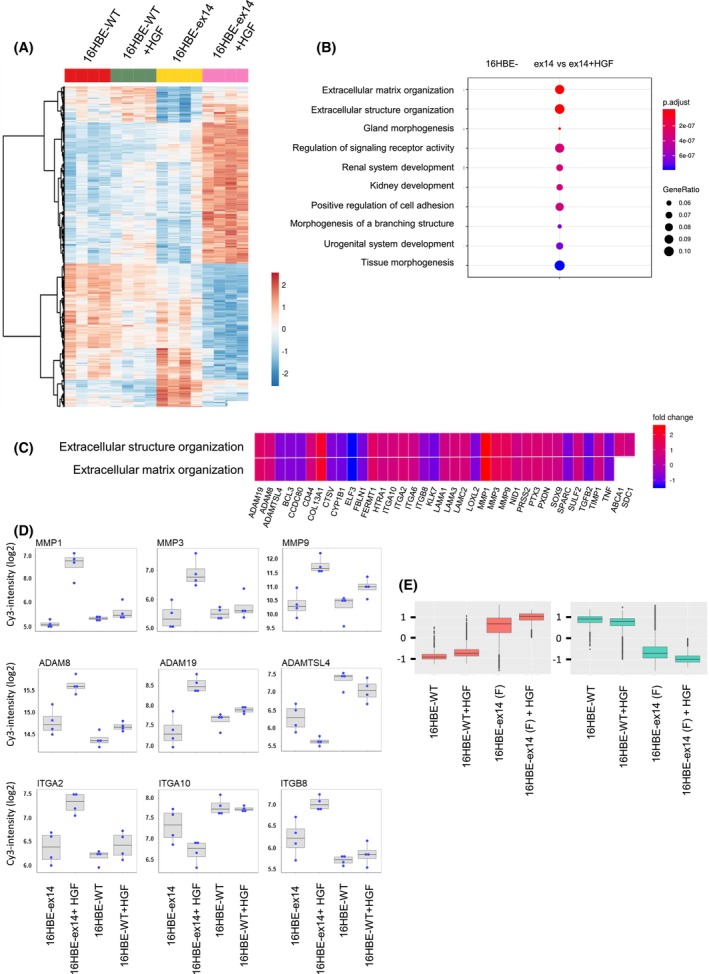
Heat map and gene ontology (GO) enrichment of genes differentially expressed in 16HBE WT or 16HBE‐ex14 cells in response to HGF. 16HBE cells expressing either MET WT or METex14 were grown 24 h, serum‐starved 2 h and treated for 24 h with HGF (20 ng·mL^−1^). mRNA was extracted and gene expression determined by DNA microarray. (A) Heat map of genes significantly differentially expressed in response to HGF (*P*‐value (adj) < 0.05 and absolute fold change > 1.5) between indicated conditions (*n* = 4 for each condition). Scale bar represents relative gene expression changes scaling. (B) Dot plot of GO enrichment of genes significantly differentially expressed in 16HBE cells stimulated or not with HGF according to the ‘biological process’ annotations of Gene Ontology. Dot size represents ratio of significantly differentiated genes. Colour scale represents *P*‐value (adjusted). (C) Heatmap‐like functional classification of gene list for the subgroups of ‘extracellular structure and matrix organization’ GO enrichment, with colour indicating the fold change of each gene comparing 16HBE‐ex14 cells activated with HGF to basal conditions. (D) Box‐and‐whisker plot of selection from the significantly differentiated GO ‘extracellular matrix organization’ analysis (intensity in log2) of MMP, ADAM and integrin families. All genes were significantly differentially regulated in the transcriptomic analysis of 16HBE‐WT cells and 16HBE‐ex14 cells activated by HGF. The boxplot shows the 25^th^, 50^th^ and 75^th^ percentiles while the blue points show the normalised expression values for each sample (*n* = 4 per condition). The median is indicated by the line across the box. (E) Box‐and‐whisker plot of the normalised expression of the top 100 most deregulated genes in the 16HBE‐ex14 cells activated with HGF to basal conditions. The comparison, performed for the four experimental conditions, show upregulated genes in red and downregulated genes in green.

To further characterise responses induced by MET ex14 in the absence of other oncogenic alterations, transcriptomic programs of 16HBE‐WT and 16HBE‐ex14 (F) cells were compared, with and without 24‐h HGF stimulation. This time point was selected following the observation that METex14 and downstream ERK1/2 and AKT phosphorylations was maintained after 24 h of HGF treatment (Fig. S2B). HGF stimulation induced significant differential expression of 497 probes between 16HBE‐ex14 and 16HBE‐WT cells at a fold change threshold fixed at 1.5 (absolute value) and adjusted *P*‐value below 0.05 (260 upregulated and 237 down regulated), demonstrating a strong ligand‐dependant response (Fig. 3A).

Gene enrichment by Over Representation Analysis revealed several differentially expressed genes involved in extracellular matrix organisation (Fig. 3B,C). These include matrix metalloproteases (MMP1, 3 and 9), integrins (ITGA2, A10 and B8) and ADAM proteases (ADAMTSL4, ADAM 8 and 19; Fig. 3D,E).

HGF activation induced only a small transcriptional response in 16HBE WT cells, with no genes differentially regulated at the thresholds described above (Fig. 3A). Nevertheless, most of the genes regulated by HGF in 16HBE‐ex14 cells were also identified in 16HBE‐WT cells, but at a lower range, which did not reach a *P*‐value of 0.05 (Fig. 3E).These include the integrins and proteases mentioned above.

These results indicate that, following a 24‐h stimulation, rather than differential gene targets, METex14 is stimulating the expression of the same genes as MET WT but more robustly. This suggests that HGF triggers a sustained gene transcription in METex14 cells, consistent with the observed sustained phosphorylation of MET, AKT and ERK1/2 compared with WT (Fig. 1).

### 3.4 HGF expression is detected in NSCLC patient samples with METex14 alterations

From a cohort of NSCLC patients previously identified to harbour MET exon 14 skipping alterations [47], further molecular analyses were performed with residual FFPE tumour samples from 18 patients. Clinical and molecular characteristics of the tumours, including METex14 mutations, MET expression score, *MET* gene amplification and other genetic alterations identified by CLAPv1 NSG, CGH and PTEN‐IHC [43, 47], are reported in Table S1. *MET* amplification was detected in only one patient. Other genetic alterations were detected in eight patients, including two with *TP53* mutations, two with *PIK3CA* mutations, one with activating *KRAS* mutation, one with *GNAS* mutation, two with *PTEN* loss, one with *MDM2* amplification and one with *CDK4* amplification.

**Fig. 4:**
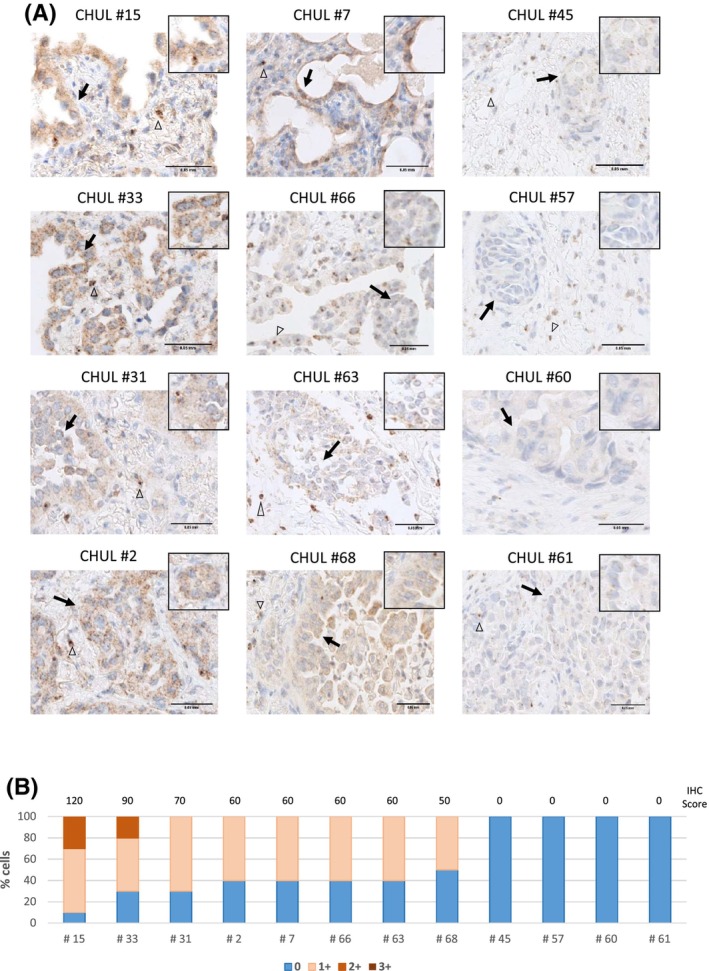
HGF expression in METex14 NSCLC patient tumours. (A) HGF immunostaining (brown) and nuclear staining with haematoxylin (blue) on FFPE tumour samples from NSCLC patients harbouring METex14 mutations. Empty arrowheads indicate examples of stromal cells known to express HGF and arrows indicate examples of tumour cells. Scale bar = 0.05 mm. (*n* = 1) (B) Samples were semi‐quantified by pathologist visual scoring of staining on a scale of 0–3+. A semiquantitative scoring was performed by multiplying the percentage of positive stained cells by the intensity of labelling.

Because HGF was required to trigger the observed biological responses in the METex14‐expressing cell model (Fig. 2), the expression of HGF was measured in the available patient samples (Fig. 4A). HGF immunostaining was considered interpretable when the proportion of tumour cells was sufficient, and positive staining was detected on stromal control cells. Using a semiquantitative scoring (see Section 2), HGF expression was detected in the cytoplasm of the tumour cells in eight of 12 interpretable samples (Fig. 4B).

Interestingly, all the tumours displaying HGF expression had high METex14 expression (IHC2+ and IHC3+) but without *MET* or *HGF* gene amplification as determined by fluorescence *in situ* hybridisation of MET (FISH) and comparative genomic hybridisation (CGH; Table S1).

These results indicate that HGF is present in two‐thirds of the patient samples with METex14 alterations analysed. Moreover, the detection of HGF in cancer cells is consistent with its requirement for METex14 activation, signalling and oncogenity, as observed in our cell lines.

### 3.5 Activation of METex14 by HGF promotes tumour growth and sensitises tumours to MET‐TKIs *in vivo*

**Fig. 5:**
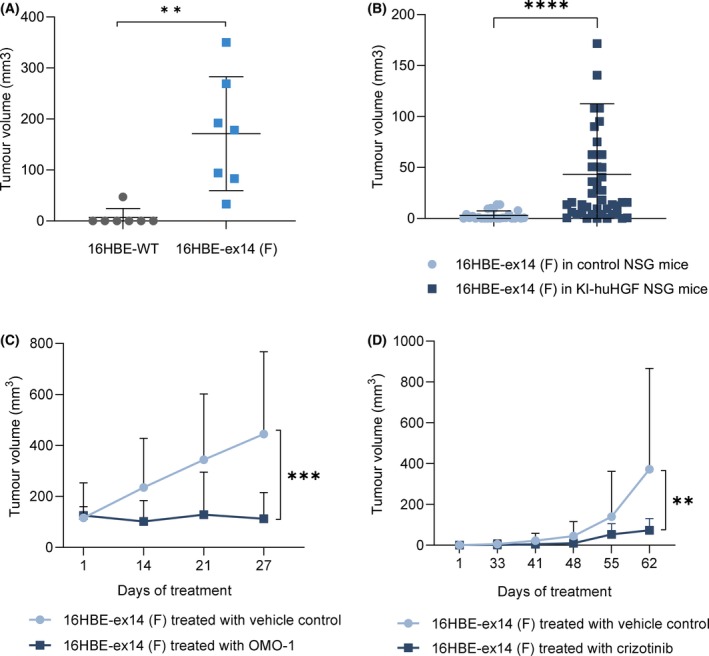
MET exon 14 loss sensitises tumours to MET‐TKIs in the presence of HGF. *In vivo* tumour xenograft experiments were performed with humanised HGF knock‐in NSG (KI‐huHGF) or control NSG mice. (A) Scatter plots of the tumour volumes (mm^3^) at end point in KI‐huHGF mice xenografted with 16HBE‐WT or 16HBE‐ex14 cells (*n* = 7 per group). (B) Scatter plots of the tumour volumes (mm^3^) 8 weeks after xenografts of 16HBE‐ex14 cells in control NSG (*n* = 26) or KI‐huHGF (*n* = 44) mice. (C) Tumour growth curves of 16HBE‐ex14 in KI‐huHGF mice treated, when tumours reached a mean volume of 100 mm^3^, with 48 mg·kg^−1^·day^−1^ of OMO‐1 or vehicle control (*n* = 8 per group). (D) Tumour growth curves of 16HBE‐ex14 in KI‐huHGF mice treated, 30 days after cell xenografts, with 50 mg·kg^−1^·day^−1^ of crizotinib or vehicle control (*n* = 14 per group). (A, B) Statistical analyses were performed with a nonparametric Mann–Whitney test. (C, D) Data are mean tumour volumes (mm^3^) of indicated number of tumours, −/+ SD. Statistical analyses were performed with a two‐way ANOVA test to compare the whole curves. **P ≤ 0.01, ***P ≤ 0.001, ****P ≤ 0.0001.

To investigate METex14‐versus MET WT‐dependent growth *in vivo*, tumour xenografts were performed in humanised HGF knock‐in NSG mice to allow the activation of MET in the human‐derived CRISPR 16HBE cell lines. Ten weeks after injections, only one KI‐huHGF mouse of seven (14%) mice implanted with 16HBE‐WT cells formed tumours, with a tumour volume of 47 mm^3^. By contrast, all seven mice implanted with 16HBE‐ex14 cells formed tumours, with a mean volume of 171 mm^3^ (Fig. 5A). Thus, the mean volume of tumours formed by cells expressing METex14 was significantly higher than the volume of MET WT tumours (*P* < 0.01), indicating that exon 14 splicing drives MET oncogenicity in KI‐huHGF mice.

To assess the requirement of HGF for METex14 tumorigenesis, in a second experiment, both NSG control and KI‐huHGF mice were injected subcutaneously with 16HBE‐ex14 cells. Eight weeks after injections, only nine of 26 xenografts (35%) in control mice generated tumours, while 38 of 44 xenografts (86%) in KI‐huHGF mice generated tumours. The mean volume of tumours formed by cells expressing METex14 in KI‐huHGF mice was significantly higher than the volume of tumours in NSG control mice (*P* < 0.001). Thus, only nine of 26 xenografts (35%) in control mice generated tumours, which in addition were small. Conversely, 38 of 44 xenografts (86%) in KI‐huHGF mice generated tumours, which were larger (Fig. 5B).

KI‐huHGF mice implanted with 16HBE‐ex14 cells were then followed until tumours reached 100 mm^3^ and treated with the novel, orally available MET‐TKI OMO‐1 or the vehicle control [48]. As shown in Fig. 5C, OMO‐1 efficiently blocked tumour growth compared with mice receiving the vehicle control (*P* < 0.001). In a similar manner, the established and clinically relevant MET‐TKI crizotinib [49] significantly delayed and reduced tumour growth when used to treat KI‐huHGF mice 30 days after implantation of 16HBE‐ex14 cells (Fig. 5D).

These results demonstrated that METex14 is oncogenic *in vivo*, in the absence of other genetic alterations or MET overexpression, and that its oncogenicity is greatly increased in presence of human HGF. They also show that HGF‐activated METex14 driven tumours are sensitive to MET‐targeted therapy.

## 4 Discussion

MET exon 14 skipping alterations occur in approximately 3–4% of patients with NSCLC, and MET‐targeted therapies, including the recently approved tepotinib and capmatinib, are being investigated in these patients. However, objective response rates achieved with MET‐TKIs in this setting is strikingly lower than those observed with TKIs in other models of oncogene addiction in lung cancer [50, 51]. METex14 has been found to co‐occur with a range of other alterations including those affecting MET directly, such as gene amplification or protein overexpression or other driver oncogenic mutations such as of PI3K or KRAS [47, 52, 53, 54, 55]. Whether such alterations are necessary contributors of METex14 driven tumorigenesis and response to MET inhibitors has remained unclear [47, 52, 53, 55]. In order to better predict response to MET‐targeted therapies in patients, it is important to understand whether exon 14 skipping alone can lead to MET addiction, drive tumorigenesis and sensitise patients to MET‐TKIs. Moreover, the requirement of HGF for the oncogenicity of METex14, especially *in vivo*, remained unclear with the report of various models which did not clearly address this question. Thus, an in‐depth characterisation of the factors that can affect METex14 tumorigenesis and activity of MET inhibitors is required to improve patient treatments and outcomes. Here we show, by genome editing of the nontransformed lung epithelial cells 16HBE, that HGF‐activated METex14 when expressed at the endogenous level, in the absence of *MET* amplification or other genetic alterations, transforms these cells *in vitro* and, importantly, induces tumour formation.

In other models of oncogene addiction, the mutant driver activity is usually independent of ligand stimulation. For example, mutations in the kinase domain of EGFR, induce a constitutive activation of the kinase activity through a conformational change of the ATP‐binding pocket. As a result, the activation of EGFR and downstream signalling is not dependent anymore on ligand binding [2]. Rearrangements affecting other receptor tyrosine kinases, such as ALK, usually lead to the loss of the extracellular, ligand‐binding, domain and transmembrane domain, resulting in the cytoplasmic localization of the chimeric protein [56]. The METex14 mutation is a one‐of‐a‐kind alteration, since it affects neither the extracellular domain nor the kinase domain and does not induce constitutive activation of the kinase domain. Instead, as METex14 leads to the loss of the juxtamembrane domain, and thus the loss of the ubiquitin‐ligase Cbl docking site Y1003, the oncogenicity of METex14 has been attributed to its increased stability [20, 25, 57], which we have also observed in the transformed H596 cells expressing endogenous METex14 (Fig. 1B,G). However, in the nontransformed 16HBE cells, METex14 degradation was not significantly impaired upon HGF stimulation (Fig. 1A,E). A similar observation was made in AALE, another model of non‐transformed epithelial cells [37], suggesting that, in the absence of any other genetic alteration, METex14 oncogenicity does not result from an increased stability and that it can compensate for a lack of Cbl binding, leading to degradation.

While some studies have reported constitutive activation of METex14, leading to its oncogenecity and cell transformation, their cell models have relied on concomitant MET overexpression [13, 25]. Our isogenic cell model, in which endogenous MET was edited with no concomitant overexpression, has allowed us to clarify the crucial need of HGF cooperation to trigger METex14 oncogenicity *in vitro* and, moreover, *in vivo*.

Our results are consistent with previous reports of METex14 oncogenicity *in vitro* [25, 31, 37]. Interestingly, they include another isogenic AALE human immortalised bronchial epithelial isogenic cell model suggesting METex14 sustained signalling and demonstrating enhanced anchorage independent growth upon HGF stimulation [37]. Our experiments clearly demonstrate that HGF stimulates more robust and sustained METex14 activation and signalling (Fig. 1), increased spheroid viability, cell migration and invasion, reduced cell contacts in low‐density colonies (Fig. 2), robust gene transcription (Fig. 3), *in vivo* tumorigenesis (Fig. 5A,B) and response to MET‐TKI (Fig. 5C,D). Thus, we demonstrate for the first time the growth of nontransformed human lung cells expressing METex14 in mice in an HGF‐dependent manner. As murine HGF is unable to activate human MET [35, 36], our isogeneic model grafted in NSG mice humanised for HGF is a unique valuable preclinical *in vitro* and *in vivo* isogenic model that closely approximates the physiological condition, and which may be used to further characterise the mechanism of METex14 oncogenicity.

We have observed that unstimulated 16HBE‐ex14 cell numbers was modestly increased at 72 h of culture in 2D as compared to 16BE‐WT (Fig. 2A) and had several genes upregulated or downregulated (Fig. 3), compared with 16HBE‐WT cells. However, as HGF was found required for METex14‐dependent spheroid survival and tumour growth, these results indicate that some changes already occur with exon 14 splicing in the absence of HGF although they are not sufficient to drive MET oncogenicity. Therefore, potential basal effect of METex14 was not further investigated in this study.

The results showing that HGF stimulation triggers 16HBE‐ex14 cells growth in less compact colonies when cultured in low density (Fig. 2C,D) indicate that, in these conditions, activated METex14 triggers the cells to stay apart from each other inside colonies. This could result from a molecular modification preventing cell–cell adhesion and/or enhanced motility leading to cell scattering. Cell scattering and migration are key functions ascribed to HGF, also called Scatter Factor, and believed to contribute to HGF and MET‐dependent metastatic spread [58]. Interestingly, our transcriptomic results in 16HBE‐ex14 cells have pointed to an increase in expression of a range of genes mostly involved in extracellular matrix and structure regulation upon HGF stimulation. All together, these results strongly suggest that, HGF‐activated METex14 triggers in response to its ligand, important changes that can contribute to its oncogenicity during cancer progression.

It was noted that both cell models expressing MET WT used in this study (nontransformed cells 16HBE and NSCL H226) require MET exon14 splicing to respond to HGF in signalling and/or functional assays (Figs 1 and 2). The poor responses of unmutated cells likely results from transient MET activation and downstream signalling (by contrast to 16HBE‐ex14 or H596 cells). According to the literature, several NSCLC cell lines expressing endogenous MET WT displayed a transient MET activation and signalling coupled to poor biological response [25, 31]. Within a panel of 28 non–small‐cell lung cancer cell lines expressing MET WT, the proliferation of at least 13 cells (including H226) was not or only slightly increased upon HGF stimulation and many other cells only had a mild response. H596 (expressing METex14) was the strongest responder. It is therefore possible that in many lung cells, including NSCLC, the ex14 deletion is necessary for a sustained signalling of MET and subsequent biological response.

We have also established the relevance of our experimental results using lung tumour tissues from NSCLC patients harbouring MET exon 14 skipping. In two thirds of the patient samples with interpretable HGF immunostaining, expression was detected at the surface or cytoplasm of cancer cells, in addition to the classical stromal localisation. Our results are in agreement with previous reports of intratumoral HGF expression in most lung tumour subtypes, with a strong HGF immunostaining reported in 42 to 70% of NSCLC tumours [59, 60, 61]. Additionally, elevated levels of HGF were strongly associated with poor outcomes in NSCLC patients [62, 63]. Although epithelial cells do not typically express HGF, one explanation for this finding could be that HGF transcription is switched on in cancer cells, triggering autocrine MET activation. Accordingly, a recent study has reported high levels of HGF transcripts in lung squamous cell carcinomas expressing or not METex14 [37]. It is also possible that HGF detected in the cancer cells is produced in the stroma but bound on MET at the plasma membrane or in the cytoplasm after internalisation. We have previously shown that HGF‐bound MET internalises and continues transmitting signals from endosomes [64], which can contribute to MET oncogenicity *in vitro* and *in vivo* [6]. Regardless of the mechanism involved, the presence of HGF in NSCLC tumours harbouring MET exon 14 skipping strongly indicates the presence of an HGF‐METex14 signalling axis in these patients, which may have a deep impact on a ligand‐dependent oncogene addiction. Only two patients in the cohort received a MET‐TKI; thus, a correlation between patients response and HGF positivity was not possible to assess.

Moreover our results demonstrated that, when expressed at endogenous level and in the absence of concomitant genetic alterations, the oncogenicity of HGF‐activated METex14 was efficiently impaired by various MET‐TKIs. Taken together with our experimental results, this suggests that HGF immunostaining, alongside METex14, may be assessed in future studies as a potential biomarker to predict response to MET‐targeted therapies and select patients for their use. Our results may therefore have future clinical implication.

## 5 Conclusions

We report that METex14 drives spheroid survival, cell motility and invasion and *in vivo* tumourigenesis in nontransformed human lung cells in an HGF‐dependent manner. Our CRISPR‐edited isogenic cells grafted in NSG‐hHGF knock‐in mice present a valuable preclinical model that closely approximates the physiological condition, and which may be used to further characterise the mechanism of METex14 oncogenicity and evaluate MET‐TKI treatments.

## Conflict of interest

PJ reports personal fees and non‐financial support from Novartis and Boehringer Ingelheim FRANCE, non‐financial support from Pierre Fabre, Chugai Pharma and MYLAN MEDICAL SAS, outside the submitted work. SH participates in advisory board for Brystol‐Myers Squibb and Boehringer Ingelheim. CD reports personal fees and non‐financial support from AstraZeneca, Novartis pharma SAS, Roche SAS, Boehringer Ingelheim France, Pfizer, outside the submitted work. MCC participates in advisory boards for Pfizer and Roche. ABC participated in advisory boards or received honoraria from Abbvie, Amgen, Astra‐Zeneca, Bristol‐Myers Squibb, Merck & Co, Pfizer, Roche, Novartis, Takeda, Janssen, Sanofi and received grants payed to ABC's institution from Novartis, Merck, Roche. TP headed OCTIMET Oncology NV and DeuterOncology NV. MFe, BH, SP, VG, AV, BDL, AM, GW, PG, JPM, SS, EC, JS, TS, PF, LG, MFi, DT, SK and ZK declare no competing financial interests.

## Author contributions

MFe, BH, PJ, DT, ABC, SK and ZK conceived and planned the experiments. MFe, SP, CD, LG, DT and ZK designed and created the cellular models. MFe, BH, PJ, SP, MJT, AV, AM, JS, EF, JPM, MFi, SS, KBB, MN, PF, SK and ZK performed *in vitro* experiments and processed the data. MFe, BH, PJ, AV, MN, AM, TP, DT and ZK contributed to the design of animal experiments, processed the experimental data and performed the analysis of the results. MFe, JPM, SS, DT and ZK, contributed to transcriptomic analysis under the supervision of MFi, PJ, VG, CD, SH and ZK contributed to patient samples collection and data analysis under the supervision of MCC and ABC, MFe, BH, PJ, JS, KBB, MFi, MCC, ABC, DT, SK and ZK performed data curation. MFe, BH, PJ, DT, ABC, SK and ZK wrote the manuscript with input from all authors. DT, ABC, PF, SK and ZK contributed to funding acquisition and SK and ZK directed the project.

## Supporting information


**Fig. S1.** CRISPR/Cas9 gene editing in 16HBE cells.

**Fig. S2.** Sustained downstream signalling and motility capacities of 16HBE‐ex14 clone 7 cells are dependent of HGF stimulation *in vitro*.

**Fig. S3.** Dose‐dependent activation of exon 14 spliced MET by HGF.

**Fig. S4.** Activation of exon 14 spliced MET and sustained downstream signalling in response to HGF stimulation.

**Table S1.** Clinical and molecular characteristics of 18 NSCLC patients harbouring METex14 mutations.

**Data S1.** Legends.

## Data Availability

The raw RNA microarray data generated in this study are available in the Gene Expression Omnibus under accession number GSE184514. The raw sequencing data are available on the Sequence Read Archive under accession number PRJNA842210. Other data that support the findings of this study are available from the corresponding authors upon request.
