# Supplementary figures and images for: MET exon 14 skipping mutation is a hepatocyte growth factor (HGF)‐dependent oncogenic driver in vitro and in humanised HGF knock‐in mice
Source: Mol Oncol. 2023 Jul 14;17(11):2257–74. doi: 10.1002/1878-0261.13397 (PMC10620121; doi:10.1002/1878-0261.13397)

Supplemental Figure S1

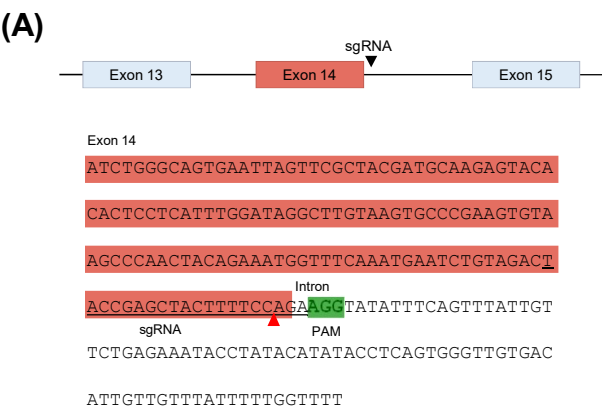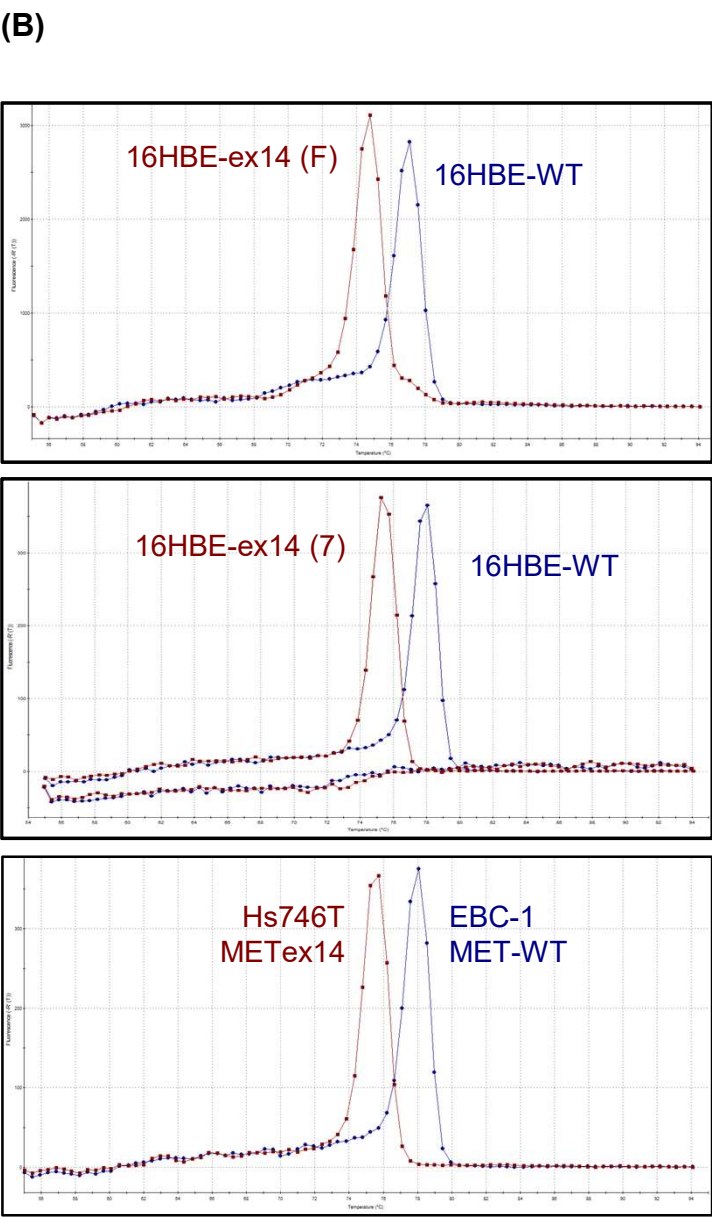

Supplement: Supplementary file 1 — Fig. S1. CRISPR/Cas9 gene editing in 16HBE cells. [file MOL2-17-2257-s004.pdf]

Supplemental Figure S2

(A)

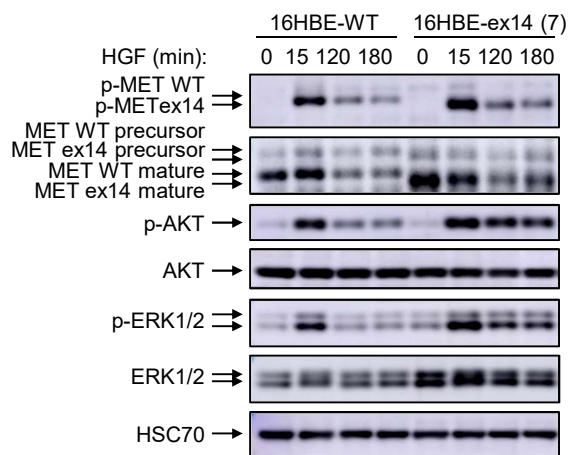

(B)

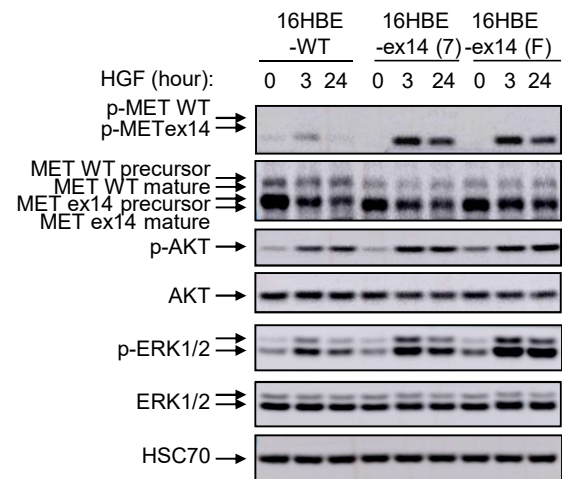

(C)

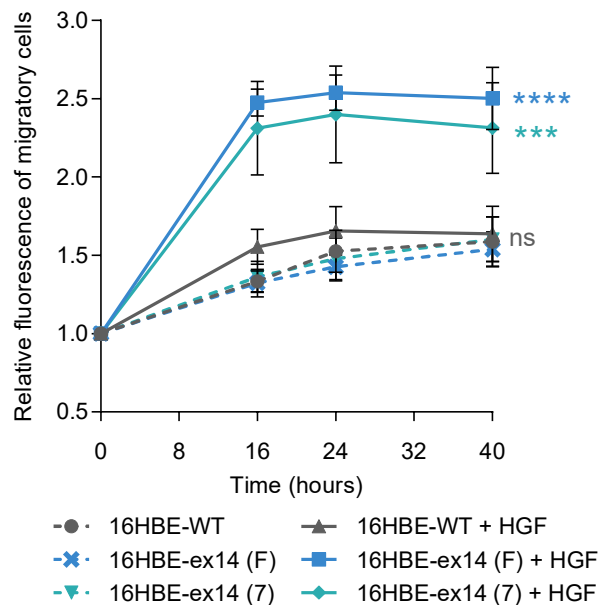

(D)

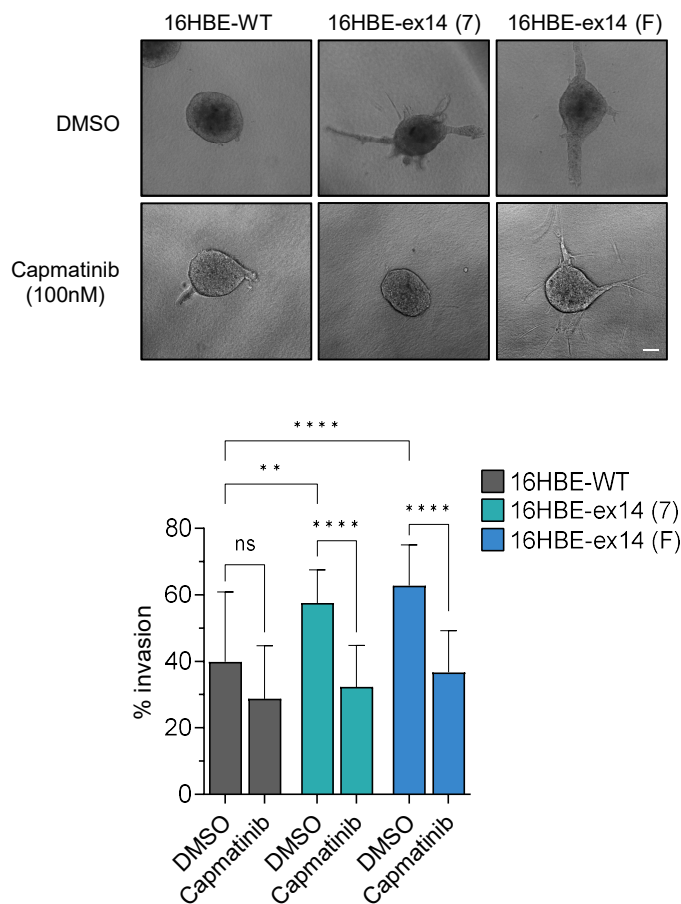

Supplement: Supplementary file 2 — Fig. S2. Sustained downstream signalling and motility capacities of 16HBE‐ex14 clone 7 cells are dependent of HGF stimulation in vitro. [file MOL2-17-2257-s001.pdf]

Supplemental Figure S3

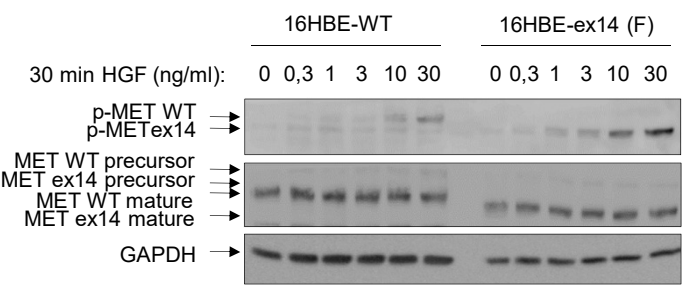

Supplement: Supplementary file 3 — Fig. S3. Dose‐dependent activation of exon 14 spliced MET by HGF. [file MOL2-17-2257-s002.pdf]

Supplemental Figure S4

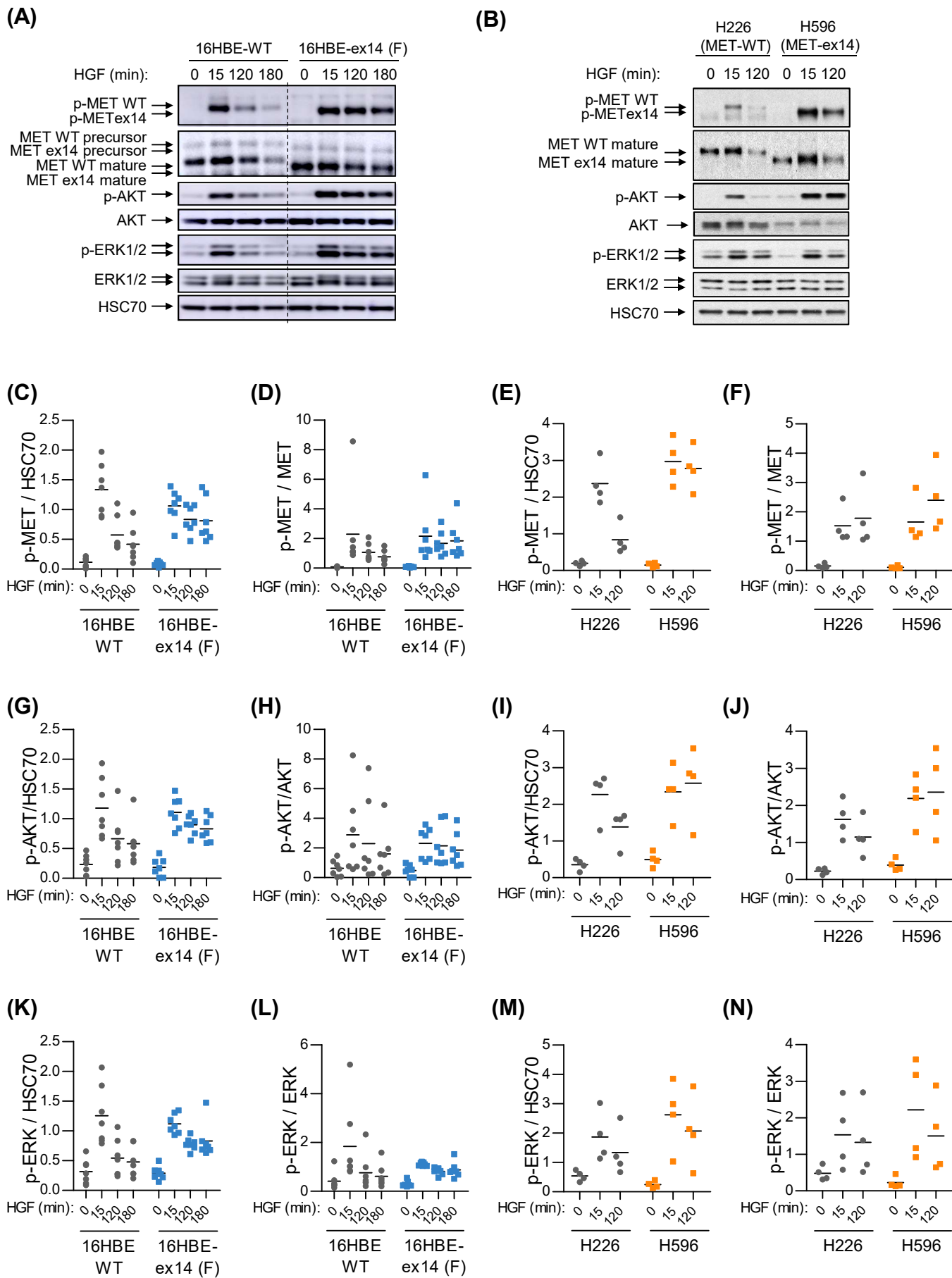

Supplement: Supplementary file 4 — Fig. S4. Activation of exon 14 spliced MET and sustained downstream signalling in response to HGF stimulation. [file MOL2-17-2257-s003.pdf]
