# Supplementary material for: MET exon 14 skipping mutation is a hepatocyte growth factor (HGF)‐dependent oncogenic driver in vitro and in humanised HGF knock‐in mice
Source: Mol Oncol. 2023 Jul 14;17(11):2257–74. doi: 10.1002/1878-0261.13397 (PMC10620121; doi:10.1002/1878-0261.13397)
Supplement: Supplementary file 5 — Table S1. Clinical and molecular characteristics of 18 NSCLC patients harbouring METex14 mutations. [file MOL2-17-2257-s005.pdf]

Supplemental Table 1

Patient number	MET exon 14 mutation	Histologic subtype	IHC MET	MET amplif.	Ratio/ mean of copy number	Other molecular alterations	IHC HGF
**CHUL #2**	c.3076_3082+2del	ADC	3+	No	1.2/3.1		60
**CHUL #7**	c.3082+2T>C	ADC	2+	No	0.36/1.67		60
**CHUL #15**	c.2942-31_2942-4del	ADC	2+	No	1.04/2.91		120
**CHUL #31**	c.2942-36_2942-4del	ADC	2+	No	0.4/1.82		70
**CHUL #33**	c.3082G>T	ADC	2+	No	0.62/3.97	TP53 c.809T>C	90
**CHUL #63**	c.3082+2T>C	ADC	ND	ND			60
**CHUL #66**	c.2942-1G>C	ADC	ND	ND		PIK3CA c.1636C>A (Q546K)	60
**CHUL #68**	c.3080A>G	ADC	3+	ND		KRAS c.35G>A (G12D)	50
**CHUL #45**	3077_3082+1del	ADC	3+	Yes	clusters in 67% of cells	PIK3CA c.1262C>T; TP53 c.396G>C	0
**CHUL #57**	c.3082+1G>A	ADC	2+	ND		GNAS c.601C>T; MDM2 and CDK4 amplification	0
**CHUL #60**	c.3082+3A>T	ADC	3+	ND			0
**CHUL #61**	c.2942-21_2942-3del	ADC	2+	ND			0
**CHUL #9**	c.2942-14_2942-4del	ADC	1+	No	0.99/1.84	PTEN loss	NI
**CHUL #14**	c.2942-47_2949del	ADC	1+	No	0.95/1.5	PTEN loss	NI
**CHUL #37**	c.2942-19_2942-4del	ADC	ND	ND		TP53 c.610G>T	NI
**CHUL #51**	c.2942-21_2945del	ADC	2+	ND			NI
**CHUL #69**	c.2942-21_2942-8del	ADC	ND	ND			NI
**CHUL #70**	c.3082G>C	ADC	3+	ND			NI
