# Supplementary material for: MET exon 14 skipping mutation is a hepatocyte growth factor (HGF)‐dependent oncogenic driver in vitro and in humanised HGF knock‐in mice
Source: Mol Oncol. 2023 Jul 14;17(11):2257–74. doi: 10.1002/1878-0261.13397 (PMC10620121; doi:10.1002/1878-0261.13397)
Supplement: Supplementary file 6 — Data S1. Legends. [file MOL2-17-2257-s006.docx]

**Supplemental Figure legends :**

**Figure S1: CRISPR/Cas9 gene editing in 16HBE cells.**

(A) A single targeting sgRNA (underlined) was used to target the 3’ end of exon 14 (red highlighting) and splice donor site in 16HBE cells. (B) Melting curve analyses of exon 14 amplicons generated by fluorescence reverse-transcription PCR from CRISPR/Cas9 edited cell mRNA. Results for 16HBE-ex14 Clone F (Top panel) and Clone 7 (Middle panel) are displayed. Hs746T (METex14) and EBC-1 (MET WT) cells were used as controls (Bottom panel).

**Figure S2: Sustained downstream signalling and motility capacities of 16HBE-ex14 clone 7 cells are dependent of HGF stimulation *in vitro*.**

(A,B) 16HBE cells expressing either WT MET or METex14 were treated with 50 ng/ml HGF for 0, 15, 120 and 180 minutes (A) or 0, 3 and 24 hours (B). Cells were grown for 48 (A) and 24 (B) hours and serum-starved 1 hour before HGF treatment. For each condition, whole cell lysates were resolved by SDS-PAGE and analyzed by western blotting with the indicated antibodies. Data shown are representative of three independent experiments. (C) Migration of DilC12-labeled 16HBE cells, WT MET or METex14 (clones F and 7) was determined in a transwell assay, with or without HGF (20 ng/ml) in the medium of the lower chamber. Fluorescence of migrating cells was measured over time and normalised to the fluorescence at time 0 set as 1 for each cell line. Data are means of three independent experiments with three wells per condition. (D) Spheroid invasion assay. 16HBE WT, -ex14 clone 7 and -ex14 clone F cells and HGF-secreting MRC5 were co-cultured in methylcellulose hanging drops to form spheres. Spheres were subsequently placed in Collagen: Matrigel hydrogels and cultured for 3 days in presence of DMSO or Capmatinib (100nM). Brightfield representative pictures of spheroids at 3 days in the gels are represented and the percentage of relative spheroid invasion calculated from three independent experiments with at least 6 spheroids per condition for each experiment. For C and D, two-way ANOVA test -/+ SD, **p<0.01, ***p<0.001, ****p<0.0001, ns = not significant. Bar 100 µm.

**Figure S3: Dose dependent activation of exon 14 spliced MET by HGF.** 16HBE cells expressing either WT MET or METex14 were grown for 24 hours and then serum-starved overnight before treatment with a range of HGF concentrations (0 to 30 ng/ml) for 30 minutes. For each condition, whole cell lysates were analyzed by western blotting with anti-MET and anti-phospho-MET antibodies. GAPDH was used as a loading control.

**Figure S4: Activation of exon 14 spliced MET and sustained downstream signalling in response to HGF stimulation.** (A, B): 16HBE WT and METex14 (F) cells (A) and H226 (WT MET) and H596 (METex14) (B) were treated with 50 ng/ml for the times indicated. Cells were grown for 48 hours (A) and 24 hours (B) and serum-starved 1 hour before HGF treatment*.* For each condition, whole cell lysates were resolved by SDS-PAGE and analyzed by western blotting with the indicated antibodies. Quantification of phospho-signals from A and B by densitometry (Image J), normalised to a loading control (HSC70) (C, E, G, I, K, M) or to total expression of the corresponding protein (D, F, H, J, L, N). Data are represented as dot plots of seven (A) and four (B) independent experiments. The horizontal bar is the mean and each dot is the value obtained for one experiment.

**Table S1: Clinical and molecular characteristics of 18 NSCLC patients harboring METex14 mutations.** *MET* mutations leading to exon 14 skipping were diagnosed on FFPE tumor samples with the targeted CLAPv1 NGS Panel. The histological type was determined after tumour haematoxylin/eosin staining. MET expression and *MET* gene copy number was determined by IHC and FISH respectively. Other molecular alterations were determined by CLAPv1 NGS and CGH. HGF and PTEN expression was determined by IHC. (ADC=Adenocarcinoma; Neg=negative; Pos=positive; ND=not determined; NI=not interpretable).
